# A Case of Compound Pregnancy

**Published:** 1859-11

**Authors:** M. Stahl

**Affiliations:** Hartford City, Ind.


THE CHICAGO

MEDICAL JOURNAL.

VOL. II. NOVEMBER, 1859. No. 11.

### ORIGINAL COMMUNICATIONS.

ARTICLE I.

### A CASE OF COMPOUND PREGNANCY.

REPORTED BY DR. M. STAHL, OF HARTFORD CITY, IND.

Among the rare cases of obstetrics is the following, which occurred four miles from this place, to which I was called, in consultation with Dr. Clouser. Mrs. H., a respectable lady, was in her third utero-gestation, and enjoying her usual good health, when, on the 13th day of June, after a little more than ordinary exercise, she was taken sick. Natural labor pains came on, and in eight hours from the commencement of labor, she was delivered of a male child, weighing only three pounds. The nails, hair, adipose matter and muscular system were developed as at full term. The placenta and membranes immediately preceded the birth of the infant.

One child and secundines were now expelled, but the prominence of the abdomen being only partially reduced, suspicions were entertained of another fœtus in utero. Expecting pains soon to recur for the expulsion of the second fœtus, but being disappointed in this, an examination per vaginam was made, and the head of another fœtus could be pretty distinctly felt.

The os uteri did not appear soft and relaxing, but rather disposed to contract. There was some hemorrhage, but not alarming, as is sometimes the case in placenta prævia; still no labor pains recurred.

It being now about four hours after the delivery of the first child, and nothing but external means having been employed to bring on labor pains, we now gave a few doses of the vinum ergotæ, with a view of producing pains for the expulsion of the second fœtus, or to check the hemorrhage.

The second indication was sufficiently met, the first was not. I remained with the woman until evening, the child being born early in the morning. The woman doing well, but no pain. Dr. Clouser visited her next day; found her comfortable. She gathered strength rapidly; was up in a few days, and in two weeks, was able to do her work *as if all was over.*

She did not express any great anxiety of mind as to her condition, expecting in a few days to be delivered of another living child, for she was sensible of its motions in utero. Forty-eight days after the first child was born, on the first day of August, she was taken sick, and speedily delivered of a *fine, healthy, female child*, weighing about eight pounds; the mother kindly recovering and nursing both children.

She knew nothing definite as to the time of conception, for her menses had not been established since her second accouchement. Each fœtus had its placenta, and enveloped in separate membranes. If both were fecundated at the same time, I think the first was expelled before term; but from its proportionate development, and the descent of the testes into the scrotum, it must have been at least eight months old when parturition took place. This would give to the second eighteen days over the usual term of gestation. More depends upon the degree of perfection which the fœtal organs have attained for external existence than upon the period of pregnancy.

The children remain healthy; the second is still the largest. They are now over two years of age, having been born in 1857.

